# Genetic structure at three spatial scales is consistent with limited philopatry in Ricord's Rock Iguanas (*Cyclura ricordii*)

**DOI:** 10.1002/ece3.5414

**Published:** 2019-07-02

**Authors:** Rosanna Carreras‐De León, Stesha A. Pasachnik, Glenn P. Gerber, Christopher P. Brooks, Ernst Rupp, Mark E. Welch

**Affiliations:** ^1^ Mississippi State University Mississippi State Mississippi USA; ^2^ Institute for Conservation Research San Diego Zoo Global Escondido California USA; ^3^ Grupo Jaragua Santo Domingo Dominican Republic; ^4^ Present address: Instituto Tecnológico de Santo Domingo Santo Domingo Dominican Republic; ^5^ Present address: Fort Worth Zoo Fort Worth Texas USA

**Keywords:** *Cyclura ricordii*, edge thinning, Mantel's test, natal philopatry, nest‐site fidelity, Ripley's *K*

## Abstract

*Cyclura ricordii* is an endemic iguana from Hispaniola Island and is threatened on the IUCN Red List. The main threats are predation by introduced mammals, habitat destruction, and hunting pressure. The present study focused on two nesting sites from Pedernales Province in the Dominican Republic. The hypothesis that natal philopatry influences dispersal and nest‐site selection was tested. Monitoring and sampling took place in 2012 and 2013. Polymorphic markers were used to evaluate whether natal philopatry limits dispersal at multiple spatial scales. Ripley's *K* revealed that nests were significantly clustered at multiple scales, when both nesting sites were considered and within each nesting site. This suggests a patchy, nonrandom distribution of nests within nest sites. Hierarchical AMOVA revealed that nest‐site aggregations did not explain a significant portion of genetic variation within nesting sites. However, a small but positive correlation between geographic and genetic distance was detected using a Mantel's test. Hence, the relationship between geographic distance and genetic distance among hatchlings within nest sites, while detectable, was not strong enough to have a marked effect on fine‐scale genetic structure. Spatial and genetic data combined determined that the nesting sites included nesting females from multiple locations, and the hypothesis of “natal philopatry” was not supported because females nesting in the same cluster were no more closely related to each other than to other females from the same nesting site. These findings imply that nesting aggregations are more likely associated with cryptic habitat variables contributing to optimal nesting conditions.

## 1 INTRODUCTION

“Philopatry” is a behavior defined by Mayr (1963) as a tendency for some individuals to remain in their native locality or to return to it during one or more key life history events. This behavior has been observed in many vagile animal species (Bowen et al., 2004; Brown & Shine, 2007; Freedberg, Ewert, Ridenhour, Neiman, & Nelson, 2005; Hueter, Heupel, Heist, & Keeney, 2005). Philopatric behavior can also be more extreme in oviparous species. Hendrickson (1958) proposed the “natal homing” model (also called natal philopatry and nest‐site philopatry) to explain the fidelity that marine turtles exhibit toward their native nesting sites. More specifically, natal philopatry in this study refers to the tendency of a female to return to nest at the exact site where she hatched. “Nest‐site fidelity” is a more general behavior in reptiles and is defined as the tendency of a female to return multiple times to the same exact geographic location to nest regardless of where she hatched. Individuals that present natal philopatry also exhibit nest‐site fidelity, whereas the opposite is not necessary. The best‐substantiated examples of nest‐site fidelity and natal homing in reptiles are based on genetic and behavioral evidence from sea turtles and giant river turtles. Both may migrate hundreds to thousands of kilometers from feeding and breeding grounds to nesting sites (Bowen & Karl, 1997; Valenzuela, 2001; Valenzuela & Janzen, 2001).

The study of philopatry has important implications for the understanding of animal dispersal (Bock, Rand, & Burghardt, 1985; Bolker, Okuyama, Bjorndal, & Bolten, 2007; Chilvers & Wilkinson, 2008; Dittman & Quinn, 1996; Ruusila, Pöysä, & Runko, 2001), their nesting behavior (Bock et al., 1985; Bowen et al., 2004; Brown & Shine, 2007; Freedberg et al., 2005; Knapp & Owens, 2008; Ruusila et al., 2001), and pronounced implications for the management and conservation of endangered species (Chilvers & Wilkinson, 2008; Hueter et al., 2005; Knapp & Owens, 2008; Salinas‐Melgoza, Salinas‐Melgoza, & Renton, 2009).

Iguanas disperse after emergence, and adults are highly territorial with the most dominant individuals establishing and defending territories that monopolize the highest quality habitat, the best forage, and for males the greatest density of females during the breeding season (Pérez‐Buitrago, Sabat, & McMillan, 2010). Iguanas typically stay in or near their territories year‐round with one exception, and females may migrate several kilometers to nest sites (Knapp, Prince, & James, 2016; Rand, Font, Ramos, & Bock, 1989). Other than female migration during nesting season, no evidence of sex‐biased dispersal has been observed in iguanas (Lanterbecq et al., 2010; Steinfartz et al., 2009). In this study, our primary objective is to determine whether natal philopatry influences female migration during nesting season. The continued visitation to the same location to nest that many animals show may bring some advantages for these species' persistence as well as disadvantages if these areas were threatened in some way. These locations may be important refuges for the maintenance of these species and for successful hatching of future generations. In contrast, the disturbance or destruction of these locations may increase energetic cost to iguanas during the breeding season by interfering with mate selection and during nesting season by females, specifically necessitating broader searches for alternative nesting sites, which may not have optimal conditions leading to lower hatching success, or even extirpation of local populations.

**Figure 1:**
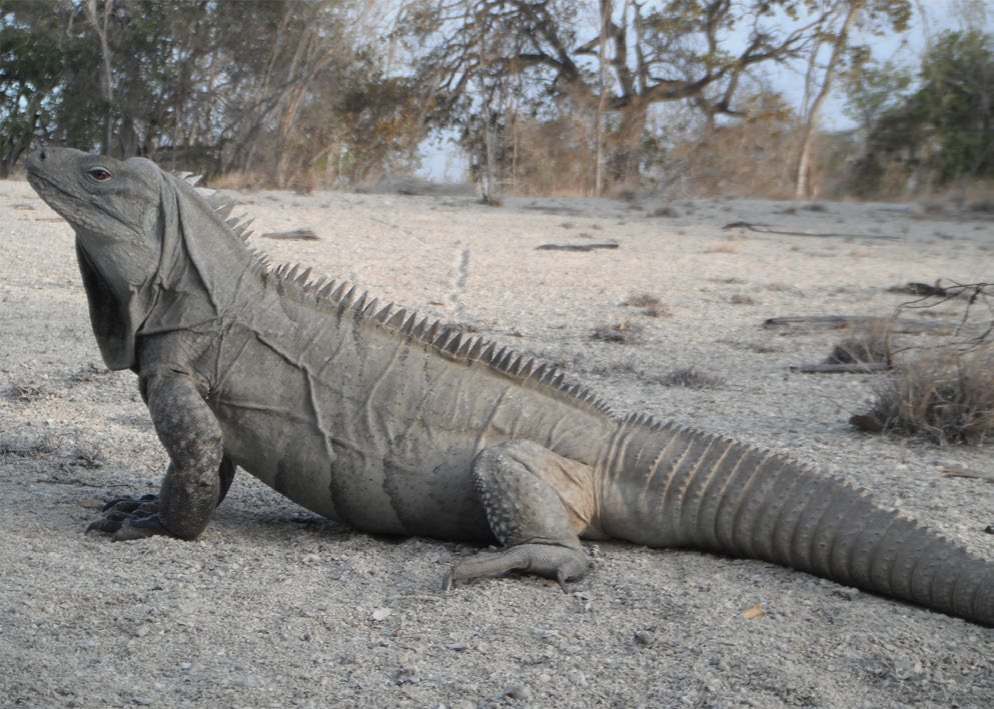
*Cyclura ricordii* in Cabritos Island, Dominican Republic

In this study, we investigated dispersal and nest‐site selection in the Endangered Ricord's Rock Iguanas, *Cyclura ricordii* (Figure 1). Species within the genus *Cyclura* often nest communally (Iverson, Hines, & Valiulis, 2004), and delineating key aspects of dispersal, site selection, and nesting behavior may enhance conservation strategies to protect important habitats and identify potential sites to incorporate into yearly monitoring programs. We used neutral molecular markers to study aspects of the nesting behavior that are difficult to characterize through observation alone.

**Figure 2:**
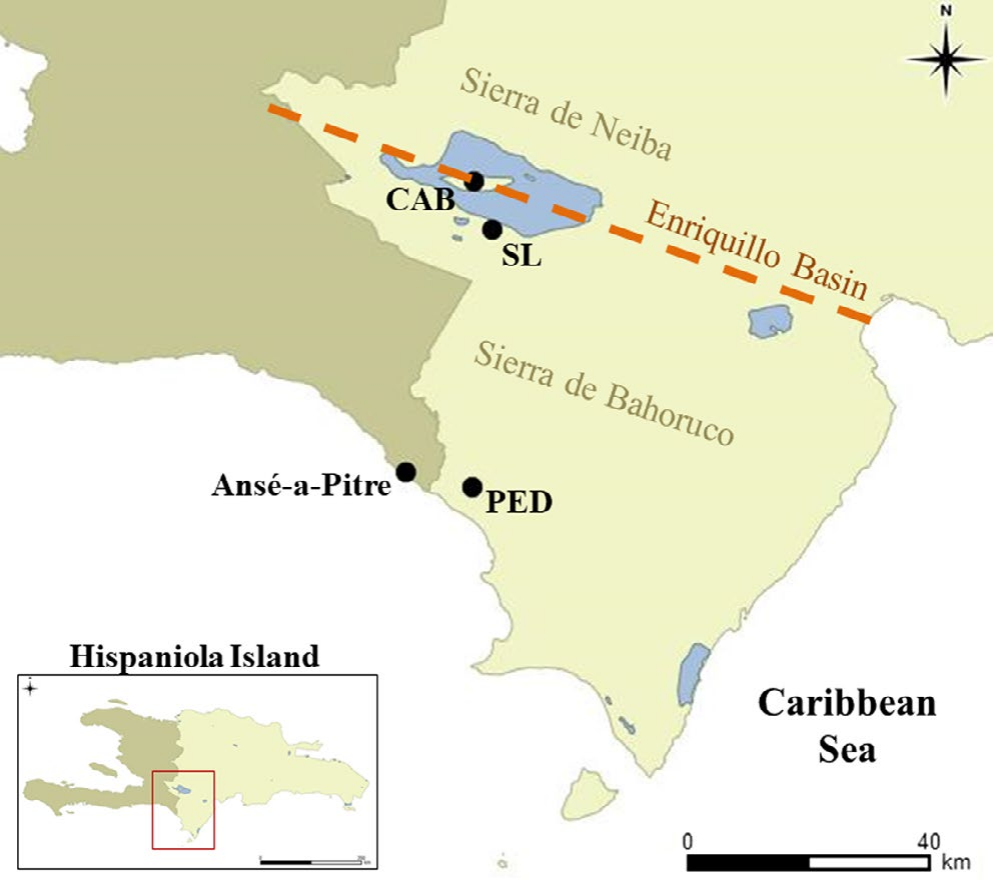
Map of the southwestern region of the Dominican Republic denoting the known locations for *Cyclura ricordii*. Sierra de Neiba and Bahoruco represent the two main mountain systems in the area. Blue shade represents water. CAB, Cabritos Island; PED, Pedernales; SL, South of Lake

*Cyclura ricordii* is an endemic vertebrate on Hispaniola, the only island in the Caribbean where two species of *Cyclura* (*C. ricordii* and *C. cornuta*) can be found in sympatry. These species are the largest native herbivores on the island, and they fulfill a crucial ecological role in their dry forest ecosystems as seed dispersers, and by contributing to nutrient cycling through foliage grazing (Hartley, Glor, Sproston, Powell, & Parmerlee, 2000; Iverson, 1985; Zoológico Nacional de la República Dominicana [Zoodom] et al., 2002). *Cyclura ricordii* is listed as Endangered according to the IUCN Red List 2019 (Pasachnik & Carreras De León in press), and only three natural populations are known in the southwestern areas of the Dominican Republic (Figure 2). An additional nest site was found across the border near Ansé‐a‐Pitre, Haiti, in 2008 (Ottenwalder, 1996; Rupp, León, Incháustegui, & Arias, 2009). The Ansé‐a‐Pitre nesting site has been formally monitored since 2010 (Rupp & Accimé, 2011). The extreme low densities of individuals sighted were attributed to intense hunting pressure in Ansé‐a‐Pitre, and further protection was recommended.

Grupo Jaragua, a Dominican NGO (www.grupojaragua.org.do), has continually monitored *C. ricordii* at all localities. The focal population under study is in the southwestern region of the Dominican Republic in Pedernales Province (Rupp, 2010). This is the only area where nesting activity has been studied over a long period of time (since 2004; Rupp, 2010). In this area, nesting occurs primarily in bottomlands called “fondos” where the iguanas excavate their retreats (Arias, Incháustegui, & Rupp, 2004). Fondos are characterized by fine, reddish, argillic soils with scrub‐like vegetation, and an open canopy (Arias et al., 2004). Four major fondos are monitored by Grupo Jaragua in the Pedernales Province.

In this study, dispersal and nest‐site selection were evaluated in two nesting sites, Tierra and Malagueta. These are the two most active nesting sites with over 100 documented nests per year at each location since 2004 (Rupp, 2010). To protect and preserve these unique areas, they were both declared as Municipal Protected Areas (EMP, *Espacio Municipal Protegido*) in the resolution 05‐2005 of the Pedernales Province City Hall (Rupp et al., 2009). These sites differ slightly in their nesting dynamics largely due to differences in land use, where Tierra abuts private agricultural land, Malagueta is more isolated. These nesting sites are approximately 1.5 km apart. Attempts to develop these nesting sites for agricultural purposes were made in 2006 and 2007 (Rupp et al., 2009). Invasive cats, dogs, and cows also heavily impact both nesting sites. The feral dogs are of special concern given that they killed at least 30 iguanas between the 2012 and 2013 nesting seasons (J. L. Castillo, pers. comm.). Also, pressure from the illegal pet trade has resulted in these nesting sites becoming hunting hot spots because of the vulnerability of iguanas during the time of oviposition.

While there is limited evidence for natal philopatry in iguanids (Bock et al., 1985; Rauch, 1988), nest‐site fidelity has been observed in multiple species of *Cyclura* (Iverson et al., 2004; Knapp & Owens, 2008). Understanding the degree to which natal philopatry influences nest‐site fidelity is critical to understanding the nesting behavior observed in *C. ricordii*. Wiewandt (1982) was one of the first to anticipate natal philopatry in iguanas. There are no prior records of natal philopatry or nest‐site fidelity for either species of *Cyclura* from Hispaniola, but preliminary data for *C. ricordii* indicated a high return rate of females to the specified nesting sites, and this may be consistent with at least a limited degree of natal philopatry and nest‐site fidelity (Rupp, Incháustegui, & Arias, 2008; Zoodom et al., 2002). Field biologists have observed that some areas within nesting sites have greater nest density creating clusters of nests within each communal nesting grounds. In some of these putative clusters, multiple females have constructed nest chambers using the same entrance tunnel (E. Rupp, pers. comm.). Using camera traps, these researchers have recorded opportunistic females excavating burrows dug by other females to construct their nests. Similar behavior was reported by Rauch (1988) for marine iguanas and sighted for *Cyclura cornuta* in the nesting sites of Pedernales. Further, observations during nesting season suggest that females may be migrating from their established territories to these remnant communal nesting grounds.

Conservation planning for endangered reptiles have benefitted from the use of molecular techniques in the past (Bowen et al., 2004; Lee, Luschi, & Hays, 2007), and while observational field studies are crucial, assessing relatedness among animals via pedigree analysis in the field may be difficult, especially for long‐lived species (Bock et al., 1985; Iverson et al., 2004; Knapp & Owens, 2008; Rauch, 1988). Molecular analyses can be used to assess genetic variation, relatedness, and population structure and infer the genetic viability of an endangered species. Implementation of molecular techniques focused on genetic screening, and variability of these populations is needed if further population declines and chances of extinction are to be mitigated. Several conservation efforts have been implemented to help recover the species since 2002, when a 5‐year Recovery Plan was created (Zoodom et al., 2002), and land in these nesting sites was purchased to limit further agricultural development in 2002 (Zoodom et al., 2002) and 2012.

Here, we test whether natal philopatry influences dispersal and nest‐site selection for *Cyclura ricordii* in the Dominican Republic. At the coarsest scale, our first objective was to test whether philopatric behavior limits dispersal between Cabritos Island (CAB) and Pedernales (PED), separated by ~40 km (Figure 2). At this scale, geographic barriers (Sierra de Bahoruco and Enriquillo Lake) and habitat preferences may be the primary drivers that create population genetic signatures consistent with philopatry. At a finer scale, we test whether natal philopatry limits dispersal between Malagueta and Tierra nesting sites within Pedernales. Tierra and Malagueta are separated by as little as 1.5 km, and no geographic barriers to dispersal exist between them. If natal philopatric behavior exists at this spatial scale, significant genetic differentiation may be present due to restricted migration and gene flow between breeding adults that return to these sites to nest. At the finest scale, we test whether natal philopatry influences the location of nests within nesting sites such that nests of related individuals are clustered or aggregated.

## 2 MATERIALS AND METHODS

### 2.1 Study system

**Figure 3:**
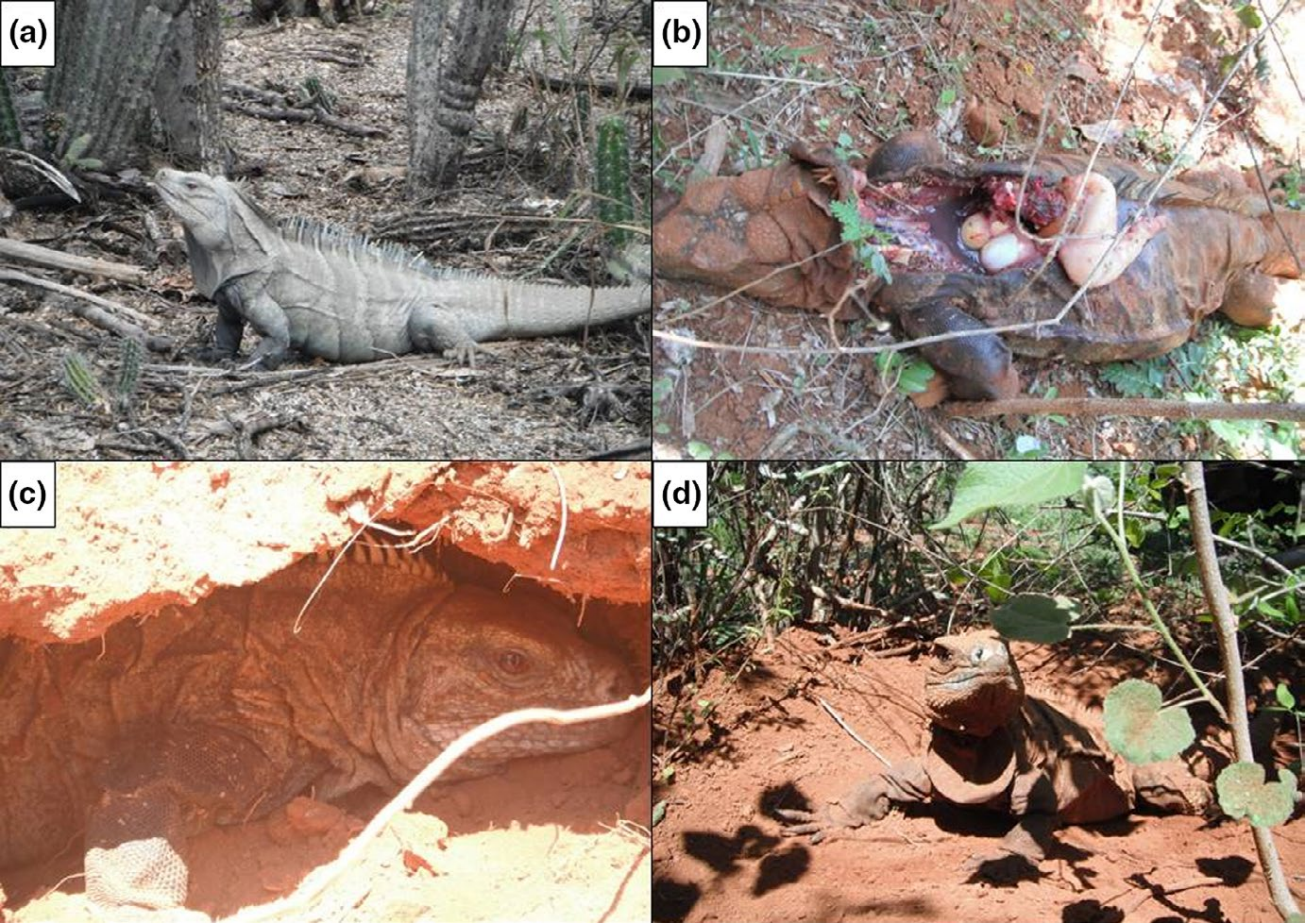
(a) An adult *Cyclura ricordii* foraging among cacti, (b) a nesting female preyed upon by a feral dog, (c) a nesting female resting while covering the nest, and (d) a nesting female completing the covering of the nest and guarding it

The genus *Cyclura*, West Indian Rock Iguanas, inhabit tropical dry forest in the Bahamas and Greater Antilles (Alberts, 1999). Species in this genus are among the world's most endangered lizards, primarily as a result of habitat degradation and the presence of exotic species (Henderson, 1992). Rock Iguanas are the largest native herbivores on many of these islands. Within the Iguanidae, 36% are known to nest communally (Doody, Freedberg, & Keogh, 2009). Iguanas in the genus *Cyclura* are iteroparous and univoltine (Alberts, 1999). However, Iverson et al. (2004) found that in *Cyclura cychlura inornata*, only one in three females nested every year. A typical nesting sequence for Rock Iguanas includes digging an entrance tunnel and chamber, laying eggs, back filling the tunnel, and defense of the nest (Figure 3; Wiewandt, 1982), although the latter is highly variable. The nesting season of *Cyclura ricordii* varies slightly from year to year; it has been reported to start as early as 8 March and continue as late as 16 June, whereas emergence occurs between 10 June and 19 September (Rupp, Incháustegui & Arias 2008; Ottenwalder, 2000). The average clutch size is 12.5 (range 2–23) eggs per nest (Rupp, Incháustegui & Arias 2008), and hatching success in Pedernales is approximately 95% on average (Pasachnik & Carreras De León in press).

Studies of other *Cyclura* (e.g., Knapp & Owens, 2008) suggest that iguanas require well‐drained soil with ample solar radiation and warm terrain for oviposition. During the hurricane season in the Caribbean (1 June through 30 November), these areas may flood periodically limiting the use of the soil for nest excavation and causing an increase in mortality of hatchlings while they are emerging from their nests (Iverson et al., 2004). West Indian Rock Iguanas, including *C. ricordii,* inhabit areas of karst limestone with limited areas of sand and soil accumulation, and nest‐site selection can be constrained by the availability of appropriate habitat (Knapp & Owens, 2008).

### 2.2 Sampling

**Figure 4:**
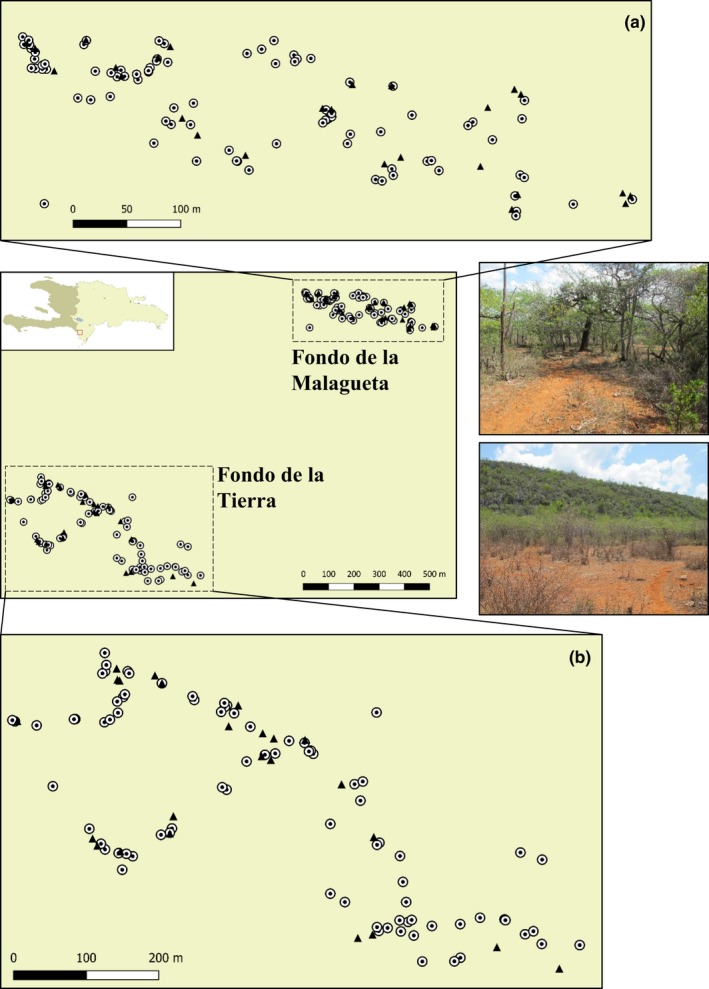
Nesting sites in Pedernales. Black triangles represent sampled nests and circles unsampled nests. (a) Malagueta nests for 2013 and (b) Tierra nests for 2013. Images to the right depict the general habitat features at these locations

Daily systematic surveys were conducted during the 2012 and 2013 nesting and hatching season across Tierra and Malagueta nesting sites in Pedernales Province, southwestern Dominican Republic. These surveys consisted of daily transects to detect and flag new nests with a number and date (Figure 4). Each nest was characterized in terms of species, and dates of egg deposition and hatchling emergence. The position of each nest was recorded with a Garmin GPS (model VISTA HCx, eTrex) using the UTM projection in the WGS84 system. Hatchlings were captured at emergence with enclosures built from metal flashing around each nest. Daily transects were done for opportunistic capture of hatchlings with a noose pole in areas where enclosures were not present. Captured hatchlings were marked by toe clipping (following Ferner, 1979 and modified by Iverson, Converse, Smith, & Valiulis, 2006), and the toe clips provided genetic samples that were stored in 95% ethanol at ambient temperature. Carcasses from unsuccessful hatching were also sampled.

Due to the logistics of this study, adult sampling was focused only in two of the four locations known for *C. ricordii*, Cabritos, and Pedernales. Adults were captured during the 2012 and 2013 field seasons using Tomahawk traps positioned in areas surrounding the nesting sites. On Cabritos Island, sampling was done using traps, noose poles, and nets. Blood was drawn from the caudal vein of adults, and tissue samples were collected from adult females that were killed by feral dogs. Blood was stored in the field at ambient temperature in SDS lysis buffer (0.1 M Tris–HCl pH 8.0, 0.1 M EDTA, 0.01 NaCl, SDS 2%; Longmire, Maltbie, & Baker, 1997).

All individuals captured and sampled were released at the site of capture. In 2012, 99 individuals were sampled: 15 adults and 84 hatchlings. In 2013, 283 individuals were sampled: 30 adults and 253 hatchlings. Only 20 of the 30 adult tissue samples yielded DNA for genetic analysis. In addition, five out of the seven samples collected from adults on Cabritos Island in 2010 were analyzed.

### 2.3 Laboratory work

Whole genomic DNA was extracted from blood and tissue samples with a Maxwell 16 Tissue DNA Purification Kit in a Maxwell® 16 MDx Research Instrument (Promega). Noncoding microsatellites were used to infer patterns of population structure and rates of gene flow. Over seventy microsatellite markers were screened and characterized for this species with the 2012 samples of hatchlings and adults (all microsatellite markers designed by Rosas et al., 2008, Welch et al., 2011, Junghwa et al., 2004, and Lau et al., 2009 were included in the screening). Amplifications were conducted with a 2,720 Thermal Cycler (AB Applied Biosystems) following standardized 3‐primer PCR amplification according to Schuelke (2000) and modified by Welch et al. (2011) in a total volume of 10 µl (i.e., 7.3 µl of ddH_2_O, 1.2 µl of master mix (10 µM of each dNTPs, 10x Tricine Taq Buffer and ddH_2_O), 0.04 µl forward primer, 0.2µl reverse primer, 0.2 µl fluorescent tagged primer, 0.4 U Taq polymerase, and 1.0 µl of DNA template). Fragment analysis was carried out at Arizona State University service laboratory, and alleles were manually annotated with Peak Scanner^TM^ Software v1.0 (Applied Biosystems).

Because mtDNA is strictly maternally inherited, an attempt was made to develop mtDNA markers to investigate sex‐specific patterns of dispersal. For this study, we surveyed a region of mtDNA bounded by ND4 and tRNA LEU for polymorphisms. Samples from Pedernales (16 individuals) and Cabritos Island (10 individuals) were sequenced according to Malone, Wheeler, Taylor, and Davis (2000) using primers ND4 and LEU (Arévalo, Davis, & Sites, 1994). There was no additional variation beyond that reported by Malone et al. (2000), and both haplotypes were observed in both populations. However, there was insufficient variation within and among populations at this locus for a meaningful analysis of fine‐scale population genetic structure.

### 2.4 Genetic analysis

genepop v. 4.2 was used to detect null alleles and to estimate gene flow (Nm) for the polymorphic microsatellites (Raymond & Rousset, 1995aa; Rousset, 2008). Loci with null allele frequencies >0.20 were removed from fine‐scale genetic analysis (Dakin & Avise, 2004). There were no significant differences when null alleles were detected using one hatchling per nest in the sampling set. We used the private allele method (Barton & Slatkin, 1986) and corrected for sample size to estimate Nm for all comparisons. Estimates of the effective population size (*N*_e_) were made using the linkage disequilibrium (LD) method (Waples & Do, 2008), the heterozygote excess method (Het_ex_; Zhdanova & Pudovkin, 2008), and the molecular coancestry method (Co; Nomura, 2008), as implemented in neestimator v2.1 (Do et al., 2014). Additional data were obtained through descriptive statistics for each locus in both adults and hatchlings from the Pedernales sites and for adults from Cabritos Island. Genetic diversity and deviations from Hardy–Weinberg equilibrium were evaluated for using arlequin v. 3.5.1.3 (Excoffier & Lischer, 2010). These include the number of alleles (*N*_a_) per locus, expected and observed heterozygosity (*H*_E_ and *H*_O_, respectively) per locus with their associated *p* values and standard deviation (Guo & Thompson, 1992; Raymond & Rousset, 1995b), and the inbreeding coefficient per locus (*F*_IS_; Excoffier, Smouse, & Quattro, 1992; Weir & Cockerham, 1984). Population‐specific *F*_IS_ indices were tested for significance with 16,000 permutations. For the adjustment of significance thresholds, a sequential Bonferroni correction was implemented (Holm, 1979).

### 2.5 Dispersal between and within geographic regions

Other F‐statistics were calculated at multiple spatial scales with an analysis of molecular variance (AMOVA) to assess genetic differentiation. *F*_ST_ was first calculated between geographic regions, Pedernales and Cabritos Island (Figure 2), to evaluate whether there is limited dispersal between these areas. A second *F*_ST_ was calculated between Tierra and Malagueta nesting sites to determine whether natal philopatry limits gene flow. All AMOVA designs and significance were calculated with arlequin v. 3.5.1.3 (Excoffier et al., 1992; Weir & Cockerham, 1984). We tested for significance across these comparisons with 16,000 permutations. To further explore genetic differentiation, we tested for differences in genetic structure over distance using Adegenet Package in r from Jombart (2008). A discriminant analysis of principal components was run (DAPC; Jombart, Devillard, & Balloux, 2010) considering all adults sampled and one hatchling per nest in the analysis.

### 2.6 Spatial structure of nests

Spatial analysis was used to test the null hypothesis that nests are distributed randomly within each nesting site. An alternative hypothesis of nonrandom distribution was also considered. To test these hypotheses, spatial data point was obtained from the monitoring program of Grupo Jaragua NGO. They provided geographic coordinates of nest positions across the years 2008–2013 for Tierra and Malagueta. All nests are within the habitat selected for the analysis which appears relatively homogenous and appropriate for nest construction. These analyses were made with the “spatstat” guide in r created by Baddeley and Turner (2014). Ripley's *K* was used to test whether nests are spatially overdispersed, clustered, or randomly distributed within nesting sites (Ripley, 1977). The function *K*(*r*) tested for a homogeneous Poisson process, assuming *complete spatial randomness* (CSR) for our dataset (Dixon, 2002). The *K*(*r*) function is:

(1.1) $$K\left(r\right)=\lambda^{-1}E$$

where *K*(*r*) refers to two‐dimensional spatial data, *λ* is the density (number per unit area) of events, and *E* refers to the number of extra events within distance *r* of a randomly chosen event (Ripley, 1976, 1977). When a Poisson process is assumed to represent CSR, the function can be written in closed form as (Dixon, 2002):

(1.2) $$K\left(r\right)=\pi r^{2}$$

Isotropy or uniformity is a key assumption of this model. For example, longitudinal and latitudinal distances should be equally correlated with density (Dixon, 2002). The function *K*(*r*) (Equation 1.2) can also be interpreted as nonstationary given that the function is defined in terms of choosing an event randomly (Dixon, 2002). The boundaries of the study area are usually arbitrary, and ignoring the influence of edge effects may produce a *K*(*r*) estimator that biases the results (Dixon, 2002). According to Dixon (2002), it is best to use the corresponding *L*(*r*) function (Equation 1.3) (Doguwa & Upton, 1989) because its variance is approximately constant under CSR (Dixon, 2002) and the function is given by:

(1.3) $$L\left(r\right)=\sqrt{\frac{K\left(r\right)}{\pi }}$$

assuming CSR, *L*(*r*) = *r*.

Significant departures between *L*(*r*) – *r* can indicate two distinct deviances from a random distribution. If *L*(*r*) – *r* < 0, then spatial data points are regularly distributed or overdispersed. If *L*(*r*) – *r* > 0, then points are underdispersed or show evidence of clustering (Dixon, 2002). Because this is inherently a two‐tailed test, the significance threshold was set appropriately (α = 0.975; Dixon, 2002). To determine statistical significance, most authors employ the Monte Carlo method (Haase, 1995). To compute statistical significance, the “envelope” command from the “spatstat” guide was used. The envelope command computed 95 simulation envelopes of the summary “Kest” (*K* estimate) to assess the goodness of fit of a point process model to the point pattern data (Baddeley, Turner, & Rubak, 2014). The lowest and highest values of *K*(*r*) (Equation 1.2) defined lower, ${\hat{K}}_{\text{lo}}$(*r*), and upper, ${\hat{K}}_{\text{hi}}$(*r*), boundaries of a 95% confidence envelope (Haase, 1995). Significant departures from these confidence envelopes indicate that a nonrandom distribution of nests may be biologically relevant.

To further evaluate the scale of aggregation or clustering, the edge thinning technique was applied (Keitt, Urban, & Milne, 1997). We assign nodes that refer to the spatial data points (nests) to specific aggregations or clusters and estimated the minimum distance, *r*, at which these nodes are considered connected. The value of *r* is iteratively increased until the entire system forms a single, connected cluster (Brooks, 2006). The plots will have “plateaus” that represent distances where little or no change in the spatial pattern occurs. The minimum distance at which each of these plateaus occurs is where the spatial structure will be minimally connected (Brooks, 2006). These minimum distances will be referred to as “threshold distances” (TD) from this point forward.

If clustering is present, then there should be an overrepresentation of short and long edges. There should also be an underrepresentation of edges with intermediate length. Each year was evaluated individually to study time–space structuring pattern. With the spatial coordinates from 2013, edge thinning technique and molecular data were joined to further evaluate spatial structuring within each nesting site. If significant clustering is observed, then the presence of natal philopatry and nest‐site fidelity could be one of the factors causing this pattern. Other influences on nest‐site selection such as habitat quality and imprinting of individuals to a particular microhabitat site could also generate a similar pattern. To test the natal philopatry hypothesis, we determined whether genetic differentiation among nests within nest aggregations was smaller than between nests in different aggregations within each fondo. If habitat quality is responsible for nesting aggregations within fondos, then the presence of clustering and the absence of fine‐scale genetic structuring would be more likely.

### 2.7 Spatial genetic structure of nests

A hierarchical AMOVA was performed to detect differentiation at a finer spatial scale within each nesting site to test the natal philopatry hypothesis. We determined whether genetic differentiation among nests within nest clusters was smaller than between nests in different clusters within each nesting site. The proportion of genetic variance attributable to clusters within nesting sites was estimated and tested for significance with 10,000 permutations across all comparisons. The variance components were defined in four hierarchic levels: (a) among clusters (*F*_CT_), (b) among nests within clusters (*F*_SC_), (c) among individuals within nests (*F*_IS_), and (d) within individuals (*F*_IT_). These variance components were assessed with Arlequin v. 3.5.1.3 (Excoffier & Lischer, 2010). Arlequin implements Wright's fixation index (*F*_ST_) to describe the amount of genetic variation for each hierarchic level according to Weir and Cockerham (1984). For Tierra, clusters created with threshold distances of 40 m and 60 m were tested, and for Malagueta, 30 m threshold distance was used.

### 2.8 Testing the isolation‐by‐distance model

A second approach tested the natal philopatry hypothesis with an isolation‐by‐distance model (Wright, 1943) using the Package adegenet in r (Jombart, 2008; Jombart & Ahmed, 2011). Mantel's test estimates the significance of correlation between distance matrices (Mantel, 1967). The “mantel.randtest” function in r was used to perform a Mantel test between two distance matrices. To create the pairwise genetic distance matrix, a dependent matrix of individual pairwise Nei's genetic distances (Nei, 1972, 1978) using the R function “dist.genpop” was created. To create a predictor matrix of pairwise linear distances in meters, we use the r function “dist” creating a pairwise individual‐by‐individual, linear geographic distance matrix from X (longitude) and Y (latitude) coordinates from each individual. The null hypothesis was that the two distance matrices are independent. The alternative hypothesis is that of a positive association between the two distance matrices, suggesting the presence of isolation by distance (Smouse, Long, & Sokal, 1986). Two assumptions were made: first, that the conditions influencing hatchling fitness (nest quality) are stable at each nesting site and second, that the presence of nests clustering is a consequence of the preference of daughters returning to nest in the same or similar locations as their mothers.

## 3 RESULTS

### 3.1 Sampling

Two of the three populations of *C. ricordii* in the Dominican Republic were sampled, Cabritos Island (CAB) and Pedernales (PED). The Pedernales population is spread across a large area, and identifying areas with high densities of individuals has been challenging. Of the 52 total adult samples gathered from both locations, 15 (28.8%) were animals killed by dogs, 12 (23%) were captured with Tomahawk traps, and 4 (7.7%) were from animals found dead on site. The rest of the wild adult captures were made with nooses and nets (21 individuals, 40.4%). The sampling success reflects the high level of threat these iguanas face, given that most of the adults sampled were carcasses.

A total of 26 nests from Malagueta and 23 from Tierra were sampled during 2013, and the number of individuals captured per nest ranged from 1 to 23. Some individuals were removed from the dataset because of poor DNA quality (i.e., carcasses of hatchlings found while opening the nest and from hatchlings that died before hatching). Twenty‐three nests from Malagueta and 22 nests from Tierra were used for analysis, and the number of individuals per nest used for the analysis ranged from 1 to 15. The 84 hatchlings sampled in 2012 were used for the screening of molecular markers and excluded from further analysis, because they only represented a very small area of Tierra and sampling effort could not be compared with the 2013 sampling.

### 3.2 Microsatellite data

Samples were genotyped at 14 microsatellite loci found to be variable for *C. ricordii*. The polymorphic markers used were as follows: Ccste05 (Rosas et al., 2008); CIDK135, CIDK144, and CIDK184 (Welch et al., 2011); Z106, Z148, Z154, and Z494 (Junghwa et al., 2004); and D1, D11 D101, D110, D111, and D140 (Lau et al., 2009). CIDK135 locus (Welch et al., 2011) was eliminated from further analysis due to high null allele frequency *p = *0.48 (Dakin & Avise, 2004; Table 1).

**Table 1:** Polymorphic microsatellite markers used in this study. Identifier (No.), locus name (Locus), and null allele frequency (p) for all individuals combined

No.	Locus	*N*_a_	Null allele frequency (p)	No.	Locus	*N*_a_	Null allele frequency (p)
1	Ccste05	10	0.06	8	Z494	7	0.08
2	CIDK135	6	**0.48**	9	D1	6	0.06
3	CIDK144	4	0.08	10	D11	14	0.05
4	CIDK184	6	0.13	11	D101	8	0.03
5	Z106	6	0.13	12	D110	10	0.11
6	Z148	6	0.07	13	D111	12	0.12
7	Z154	9	0.08	14	D140	16	0.13

Note

In bold, CIDK135 with a null allele frequency >0.20. Number of alleles across all loci and individuals (*N*_a_)

### 3.3 Genetic variation and dispersal between geographic regions and between nesting sites

The number of alleles per locus (*N*_a_) ranged from 4 to 16 for the thirteen markers across all samples (Table 1). *F*_IS_ population‐specific indices were significant for Pedernales and Cabritos Island populations when all individuals were included (adults and hatchlings) and when only adults were considered only for the Pedernales population (Tables 2 and 3). For these populations, a significant excess of homozygotes was evidenced. The degree of homozygosity for Pedernales decreases greatly when all hatchlings were included. When one hatchling per nest was used, to remove any possible biases associated with using related individuals in the Pedernales dataset, homozygosity increased and was significant. Pedernales hatchling data were partitioned by nesting sites. *F*_IS_ indices per nesting site also suggest an excess of homozygotes for hatchlings from both nesting sites, but it is only significant for Tierra (Table 3). This homozygosity level increased when only one hatchling per nest was tested for Tierra. The numbers of migrants (Nm) were evaluated between geographic regions and between nesting sites. Gene flow between Cabritos and Pedernales populations considering only adult samples was below 1 (Nm = 0.99) suggesting isolation between these populations. For the estimation of gene flow between Malagueta and Tierra, the value exceeded 1 (all hatchlings, Nm = 8.38; one hatchling per nest, Nm = 5.35), suggesting gene flow between nesting sites is high. The level of gene flow between nesting sites indicates that iguanas may be moving freely between nesting sites. Parametric estimates of effective population sizes (*N*_e_) using the LD and the Coancestry methods for both populations range between 3.5 and 105.7, and 95% confidence intervals were highly variable (Table 4), whereas for the results from the heterozygote excess method (Het_ex_), they were inclusive due to the limited sample size (Waples & Do, 2010; Table 4).

**Table 2:** Genetic variation by locus in sampled adults and hatchlings *Cyclura ricordii* from Cabritos Island and Pedernales populations

Locus	*N*_a_	Cabritos Island adults (*n* = 20)					Pedernales Adults (*n* = 20)				
		*H*_E_	*H*_O_	*p*‐val	*SD*	*F*_IS_	*H*_E_	*H*_O_	*p*‐val	*SD*	*F*_IS_
Ccste05	10	0.795	0.526	**0.002**	0.000	0.344	0.807	0.632	0.103	0.000	0.222
CIDK144	4	0.440	0.400	0.613	0.000	0.093	0.579	0.316	**0.021**	0.000	0.461
CIDK184	6	0.787	0.737	0.878	0.000	0.065	0.772	0.579	0.116	0.000	0.256
Z106	6	0.733	0.526	**0.037**	0.000	0.287	0.756	0.750	0.079	0.000	0.009
Z148	6	0.522	0.556	**0.027**	0.000	−0.066	0.737	0.650	0.090	0.000	0.121
Z154	7	0.683	0.316	**0.000**	0.000	0.544	0.603	0.412	**0.002**	0.000	0.323
Z494	7	0.681	0.550	**0.034**	0.000	0.196	0.659	0.550	0.262	0.000	0.169
D1	6	0.512	0.500	0.076	0.000	0.023	0.779	0.600	0.057	0.000	0.235
D11	9	0.773	0.778	**0.014**	0.000	−0.006	0.865	0.947	0.255	0.000	−0.098
D101	6	0.467	0.474	0.411	0.001	−0.016	0.687	0.556	**0.039**	0.000	0.196
D110	6	0.586	0.526	0.906	0.000	0.104	0.587	0.526	0.498	0.001	0.107
D111	11	0.825	0.500	**0.000**	0.000	0.401	0.541	0.278	**0.000**	0.000	0.494
D140	14	0.810	0.833	0.569	0.001	−0.030	0.819	0.737	0.234	0.000	0.103

Locus	*N*_a_	Tierra hatchlings (*n* = 105)					Malagueta hatchlings (*n* = 148)				
		*H*_E_	*H*_O_	*p*‐val	*SD*	*F*_IS_	*H*_E_	*H*_O_	*p*‐val	*SD*	*F*_IS_
Ccste05	7	0.698	0.660	**0.005**	0.000	0.055	0.702	0.706	**0.002**	0.000	−0.006
CIDK144	4	0.613	0.464	**0.000**	0.000	0.245	0.603	0.610	**0.000**	0.000	−0.011
CIDK184	6	0.749	0.667	**0.001**	0.000	0.111	0.788	0.757	**0.006**	0.000	0.040
Z106	5	0.726	0.725	**0.000**	0.000	0.001	0.682	0.630	**0.000**	0.000	0.077
Z148	4	0.653	0.567	**0.002**	0.000	0.132	0.698	0.750	**0.002**	0.000	−0.075
Z154	8	0.560	0.490	**0.001**	0.000	0.125	0.569	0.517	**0.007**	0.000	0.090
Z494	6	0.563	0.544	**0.017**	0.000	0.034	0.594	0.610	**0.007**	0.000	−0.027
D1	6	0.751	0.767	0.355	0.001	−0.021	0.744	0.767	**0.044**	0.000	−0.031
D11	13	0.838	0.796	**0.000**	0.000	0.050	0.798	0.826	**0.000**	0.000	−0.035
D101	6	0.708	0.647	**0.001**	0.000	0.086	0.728	0.718	**0.000**	0.000	0.013
D110	3	0.482	0.362	**0.013**	0.000	0.250	0.570	0.407	**0.000**	0.000	0.286
D111	12	0.657	0.451	**0.000**	0.000	0.315	0.257	0.150	**0.000**	0.000	0.417
D140	11	0.800	0.830	**0.014**	0.000	−0.037	0.773	0.669	**0.000**	0.000	0.135

Note

Locus name (Locus). Number of individuals (*n*). Number of alleles per sampling set (*N*_a_). Expected and observed heterozygosity (*H*_E_ and *H*_O_, respectively) (Guo & Thompson, 1992). Coefficient of inbreeding (*F*_IS_) according to Weir and Cockerham (1984). In bold significant P values. Standard deviation on the *p*‐value (*SD*).

**Table 3:** Output from arlequin v. 3.5.1.3. Population‐specific *F*_IS_ indices with 16,000 permutations

Sample	*n*	Locality	*F*_IS_ indices	*p*(Rand *F*_IS _≥ Obs *F*_IS_)
Adults	20	CAB	0.051	0.1674
	20	PED	0.120	**0.0046**
Adults + Hatchlings^a^	20	CAB	0.114	**0.0114**
	273	PED	0.056	**0.0000**
Hatchlings^b^	148	MAL	0.018	0.1479
	105	TIE	0.066	**0.0006**

Note

Sample size (*n*). In bold significant *p* values.

Abbreviations: CAB, Cabritos Island; MAL, Malagueta; PED, Pedernales; TIE, Tierra.

a Adults + Hatchlings (1 hatchling/nest) CAB *F*_IS_ = 0.114 (*p*‐val: 0.009); PED *F*_IS_ = 0.076 (*p*‐val: 0.0012).

b Hatchlings (1 hatchling/nest) MAL *F*_IS_ = 0.012 (*p*‐val: 0.4146); TIE *F*_IS_ = 0.073 (*p*‐val: 0.0578).

**Table 4:** Effective population size (*N*_e_) estimates

Population	*n*	LD	LD CI^a^	Het_ex_	Het_ex_ CI	Co	Co CI^b^
Cabritos	20	17.5	7.5–85.7	∞	∞	3.5	2.8–4.2
Pedernales	65	105.7	56.1–370.2	∞	∞	8.3	4.4–13.5

Note

Estimates using linkage disequilibrium method (LD), the heterozygous excess method (Het_ex_), and the coancestry method (Co). Confidence intervals (CI, 95%). LD and Het_ex_ methods include only alleles with frequencies greater than 0.05 to prevent biases associated with low allele sample sizes. Sample size (*n*).

a Confidence intervals were implemented using the jackknife method of Waples and Do (2008).

b neestimator (v2) implements a new jackknife method to estimate CI developed by Do et al. (2014).

An analysis of molecular variance between Cabritos (CAB) and Pedernales (PED) adults revealed that a significant portion of the differences in the genetic makeup of these two areas can be explained by relative isolation and lack of gene flow when only adults were considered (*F*_ST_ = 0.134, *p* « 0.01; Table 5). A significant, although less pronounced, degree of isolation was also found with an AMOVA apportioning genetic variance between Malagueta (MAL) and Tierra (TIE) nesting sites when all hatchlings were considered (*F*_ST_ = 0.019, *p* « 0.01; Table 5). This pattern decreased when one hatchling per nest was considered for analysis but was not significant. When adults and hatchlings were combined in the third AMOVA design, the degree of differentiation between CAB and PED increased to 15.9% (*F*_ST_ = 0.159, *p* « 0.01; Table 5). This differentiation between CAB and PED was also supported when adults and hatchlings were combined in the DAPC, where the Bayesian information criterion (BIC) value identified the presence of two well‐defined clusters for each population (Figure S1).

**Table 5:** Pairwise *F*_ST_ estimates across all loci

	*F*_ST_	*p*‐val
CAB versus PED (Adults)	0.134	**0.000**
CAB versus PED (Adults + Hatchlings)^a^	0.159	**0.000**
MAL versus TIE (Hatchlings)^b^	0.019	**0.000**

Note

In bold significant *p* values.

Abbreviations: CAB, Cabritos Island; MAL, Malagueta; PED,Pedernales; TIE, Tierra.

a CAB versus PED (adults + hatchlings [1 hatchling/nest]) *F*_ST_ = 0.149 *p*‐val = 0.000.

b MAL versus TIE (hatchlings [1 hatchling/nest]) *F*_ST_ = 0.004 *p*‐val = 0.950.

### 3.4 Spatial structure of nests

#### 3.4.1 Between nesting sites: Coarse spatial scale

**Figure 5:**
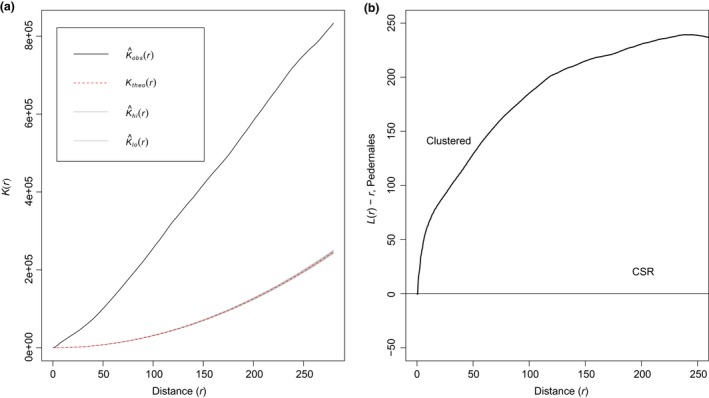
(a) Plot of *K*(*r*) versus distance (*r*) for nesting habitat in Pedernales (MAL and TIE) using the envelope function. ${\hat{K}}_{\text{obs}}$(*r*) represents the observed value of *K*(*r*) for the data, *K*_theo_(*r*) is the theoretical value of *K*(*r*) assuming a random distribution (CSR under a Poisson model), ${\hat{K}}_{\text{hi}}$ (*r*) and ${\hat{K}}_{\text{lo}}$(*r*) represent the upper and lower boundaries for the curve from 95 simulations which closely overlap with *K*_theo_(*r*). Significance level of the Monte Carlo test: 2/96 = 0.0208. (b) Ripley's *K* corresponding function *L*(*r*) – *r* plotted against distance (*r*)

Ripley's *K* was estimated at a coarse scale including data from both nesting sites. A total of 1,166 spatial points (nests) from 2008 to 2013 occurred within a 1,100 x 1,500 m rectangular plot. *K*(*r*) was estimated for distances up to 250 m. Above this cutoff distance, the spatial distribution of nests appears to be random. Observed variation in node density for all *K*(*r*) estimates, as measured by ${\hat{K}}_{\text{obs}}$(*r*), exceeded the expected variance that assumed a random distribution of points, *K*_theo_(*r*). This indicates that nests are significantly clustered when evaluated at a coarse scale (Figure 5), and within a radius of 250 m, the clustering pattern is clearly evident.

#### 3.4.2 Within nesting sites: Fine spatial scale

**Figure 6:**
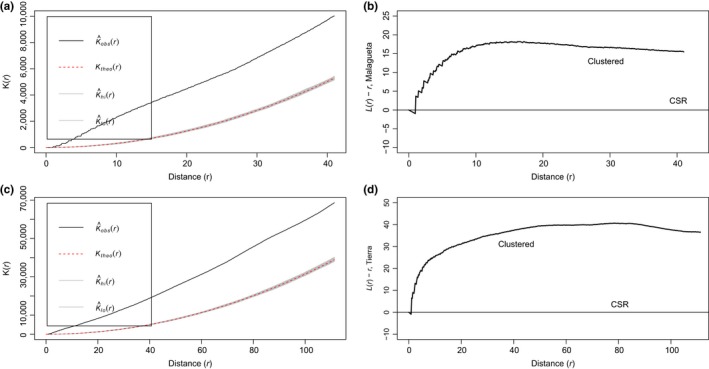
(a and c) Plots of *K*(*r*) versus distance (*r*) for (a) Malagueta and (c) Tierra using the envelope function. ${\hat{K}}_{\text{obs}}$(*r*) represents the observed value of *K*(*r*) for the data, *K*_theo_(*r*) is the theoretical value of *K*(*r*) assuming a random distribution (CSR with a Poisson model), ${\hat{K}}_{\text{hi}}$(*r*) and ${\hat{K}}_{\text{lo}}$(*r*) represent the upper and lower boundaries for the curve from 95 simulations which closely overlap with *K*_theo_(*r*). Significance level of the Monte Carlo test for Malagueta and Tierra were: 2/96 = 0.0208. (B and D) Ripley's *K* corresponding function *L*(*r*) – *r* plotted against distance (*r*) for (b) Malagueta and (d) Tierra

Ripley's *K* was estimated at a finer scale where each nesting site was evaluated individually. For Malagueta, a total of 573 spatial points (nests) from 2008 to 2013 that were within a 170 x 476 m rectangular plot were measured. *K*(*r*) was estimated for distances up to 40 m (Figure 6a). Tierra included 593 spatial points in a 454 x 695 m rectangular plot. *K*(*r*) was estimated for distances of up to 100 m (Figure 6c). Observed variation in node density for all *K*(*r*) estimates, as measured by ${\hat{K}}_{\text{obs}}$(*r*), exceeded the expected variance assuming a random distribution of points, *K*_theo_(*r*). This suggests a similar pattern as the one found at the finer scale for the nesting habitat in Pedernales, where nests are significantly clustered even when the finest scale is evaluated (within each nesting site; Figure 6b,d). This indicates that for Malagueta within a radius of 40 m, nests are significantly clustered and for Tierra, the radius where significant clustering is present is 100 m. After these threshold distances, the spatial pattern of nests within each nesting site is consistent with a random process. The corresponding function, *L*(*r*) – *r,* also suggests that the degree of clustering in relation to distance (*r*) varies between sites. Because of the preexisting clustering pattern of the nesting site at the Pedernales site and due to the significant degree of clustering observed for each nesting site individually, both nesting sites are analyzed separately from this point forward.

#### 3.4.3 Spatial structure of nests by year

**Figure 7:**
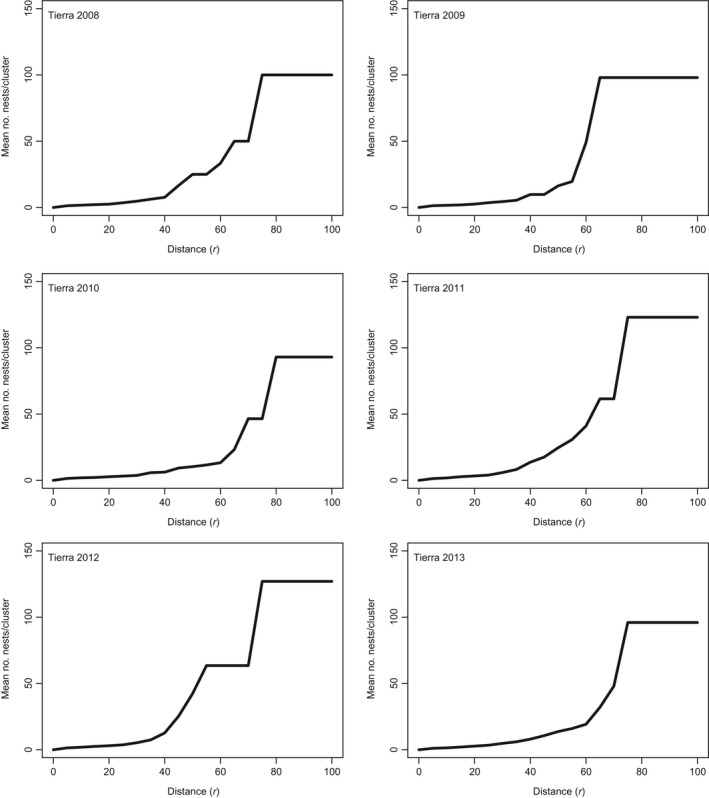
Edge thinning technique applied to Tierra by year is plotted in increments of 5 m (step command = 5 in r). Plots represent data from the *L(r)* – *r* function for *K*(*r*). Distance (*r*) is in meters. Each graph represents a different year

**Figure 8:**
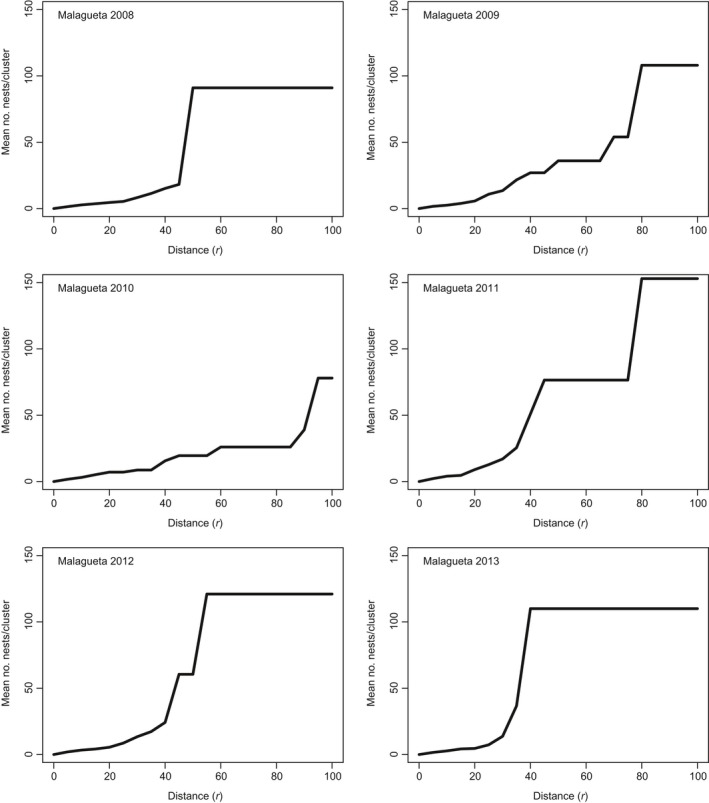
Edge thinning technique applied to Malagueta by year is plotted in increments of 5 m (step command = 5 in r). Plots represent data from the *L(r)* – *r* function for *K*(*r*). Distance (*r*) is in meters. Each graph represents a different year

Spatial points for each nesting site for every year from 2008 to 2013 were plotted to evaluate the pattern of clustering suggested by Ripley's *K* at the finest scale and to evaluate whether the clustering pattern observed changes over time. Dotted lines on Figures 7 and 8 indicate distances that were underrepresented, or where plateaus occur, and supplementary information for these plateaus is provided in Table 6. Results suggest that the scale of aggregation varies across years, as does the distance between aggregations. The range of distances at which clustering patterns were detected for Tierra ranged from 50 to 70 m and for Malagueta, these values ranged from 45 to 70 m (Table 6). If circles with radii of 50–70 m at Tierra or 45–70 m at Malagueta are considered, clustering of nests is observed over the years. For 2013, no pattern of spatial aggregation was found. All nests were part of one cluster when the edge thinning technique was used. This suggests that nests were more dispersed across nesting sites for 2013. Other cases of this dispersed pattern include Tierra in 2009 (Figure 7) and Malagueta in 2008 (Figure 8).

**Table 6:** Summary spatial pattern data from edge thinning analysis for Tierra and Malagueta across years 2008–2013

Tierra					Malagueta			
Year	Plateau ID.	Mean No. Nests/Cluster	No. Clusters	Distance (m)	Plateau ID.	Mean No. Nests/Cluster	No. Clusters	Distance(m)
2008	08‐I	25	4	50	08‐I	91	1	50
	08‐II	50	2	65				
	08‐III	100	1	75				
2009	09‐I	65	1	65	09‐I	36	3	50
					09‐II	54	2	70
					09‐III	108	1	80
2010	10‐I	46.5	2	70	10‐I	26	3	60
	10‐II	93	1	80	10‐II	78	1	95
2011	11‐I	61.5	2	65	11‐I	76.5	2	45
	11‐II	123	1	75	11‐II	153	1	80
2012	12‐I	63.5	2	55	12‐I	76.5	2	45
	12‐II	127	1	75	12‐II	76.5	2	55
2013	13‐I	96	1	75	13‐I	110	1	40

### 3.5 Spatial genetic structure of nests

Distances below the minimal plateau distance for the data points from 2013 that were obtained from the edge thinning technique were combined with molecular data. Even if plateaus were not clearly defined in the results of the 2013 edge thinning procedures and only one pattern of aggregation was observed (Figures 7 and 8), multiple distances were tested. Individuals within each putative cluster should be more closely related when compared to individuals from other clusters when assuming a philopatric behavior on behalf of related females. The distances used to subdivide each nesting site into clusters were based on results of the edge thinning analysis. For Tierra, two different threshold distances (TDs) were used: TD1 implements a distance radius of 60 m and TD2 of 40 m to detect clusters. For Malagueta, one threshold distance below the minimal plateau distance was used: TD3 implements a radius of 30 meters to detect clusters.

*F*‐statistics for each of the clustering patterns tested are shown in Table 7. Genetic structuring for the hierarchical levels (a) among clusters within nesting sites (*F*_CT_) and (c) among individuals within nests (*F*_IS_) was not significant. This indicates that the spatial genetic structuring suggested with the threshold distances used was not supported when the genetic structuring involved clusters and individuals within nests. Smaller distances were tested, and no significant differences in the AMOVA were detected. To further decrease the radii of distances used, a higher number of nests and multiple years should be included in this type of analysis. The most significant level of differentiation was (b) among nests within clusters (*F*_SC_). This result indicates that 20% of the variation in Tierra and 17.5% of the variation in Malagueta can be explained among the nests within clusters. This suggests that nests within a single cluster are not more closely related than nests from other clusters. The hierarchical level related to the degree of differentiation (d) within individuals (*F*_IT_) was moderately significant for Tierra, but not significant for Malagueta. This suggests that there is a significant deviation of alleles from Hardy–Weinberg expectations within individuals relative to the entire nesting site (Holsinger & Weir, 2009), which is consistent with the population‐specific *F*_IS_ indices estimated for Tierra (Table 3).

**Table 7:** *F*‐statistics for each of the four hierarchic levels to evaluate the clustering patterns

Hierarchical level (i–iv)	Tierra		Malagueta
	TD1: 60 m	TD2: 40 m	TD3: 30 m
*F*_CT_ =	−0.015	−0.003	0.001
*p*‐val ± *SE* =	0.887 ± 0.009	0.531 ± 0.005	0.552 ± 0.012
*F*_SC_ =	0.204	0.200	0.175
*p*‐val ± *SE* =	**0.000 ± 0.000**	**0.000 ± 0.000**	**0.000 ± 0.000**
*F*_IS_ =	−0.158	−0.158	−0.173
*p*‐val ± *SE* =	1.000 ± 0.000	1.000 ± 0.000	1.000 ± 0.000
*F*_IT_ =	0.064	0.070	0.033
*p*‐val ± *SE* =	**0.003 ± 0.002**	**0.003 ± 0.001**	0.129 ± 0.010

Note

Hierarchic levels on table: (a) among clusters (F_CT_), (b) among nests within clusters (*F*_SC_), (c) among individuals within nests (*F*_IS_), and (d) within individuals (*F*_IT_). In bold significant *p* values.

### 3.6 Testing the isolation‐by‐distance model

To evaluate natal philopatry for both Tierra (TIE) and Malagueta (MAL), a Mantel's test was performed using 13 microsatellite markers for 22 nests from Tierra and 23 nests from Malagueta where all hatchlings were included and analyses were rerun with one hatchling per nest (Table 8). When all hatchlings were included, there was a very small positive correlation for all Mantel's tests performed (Table 8). These results suggest that only 0.5% of the variation in Malagueta and none in Tierra can be explained by the isolation‐by‐distance model (Wright, 1943). This was only significant for Malagueta. When both nesting sites were evaluated together, a significant positive correlation (*p*‐val « 0.000) was still present but very little of the variation can be explained with this model (only 0.7%). When one hatchling per nest was considered, no significant correlations were detected. In this respect, the alternative hypothesis of a positive association between matrices was supported when all hatchlings were included; however, the signature of isolation by distance was lost when only one hatchling per nest was considered. In general, very little of the genetic variation could be explained by the model, which is only consistent with a limited degree of fine‐scale genetic structure.

**Table 8:** Paired Mantel test between pairwise Nei's genetic distance matrix and a pairwise geographic distance matrix for hatchlings from nesting sites from Pedernales (PED)

Item	*n*	*N*_a_	No. nests	Monte Carlo test (*R*)	*R* ^2^	*p*‐val
PED (all hatchlings)	253	91	45	0.084	**0.007**	**0.000**
PED (one hatchling/nest)	45	78	45	0.013	0.000	0.299
TIE (all hatchlings)	105	83	22	0.001	0.000	0.473
TIE (one hatchling/nest)	22	70	22	−0.119	0.014	0.857
MAL (all hatchlings)	148	83	23	0.073	**0.005**	**0.008**
MAL (one hatchling/nest)	23	71	23	−0.077	0.006	0.816

Note

Number of individuals (*n*). Number of alleles (*N*_a_). Number of permutations = 10,000. In bold significant *p* values.

Abbreviations: MAL, Malagueta nesting site; PED, Pedernales; TIE, Tierra nesting site.

## 4 DISCUSSION

We found significant differences in allele frequencies between Pedernales and Cabritos Island populations consistent with restricted gene flow between sites and small effective population sizes indicative of the magnitude of genetic drift and inbreeding that may be acting on these populations (Crow & Kimura, 1970; Wang, Santiago, & Caballero, 2016). The significant degree of differentiation observed between Pedernales and Cabritos Island populations, along with the lack of gene flow, which according to Slatkin (1987) Nm < 1 cannot counteract the effects of genetic drift, and is suggestive of nearly complete isolation between these populations (*F*_ST_ = 0.134, *p* « 0.01; Nm = 0.99), suggests limited dispersal between geographic regions for the species. This pattern of genetic structure was expected because of the magnitude of the geographic barriers between sites. The Bahoruco mountain range, which reaches almost 2,000 m asl, and Enriquillo Lake are important barriers for dispersal between Pedernales and Cabritos Island populations. Many studies have demonstrated how these types of barriers influence genetic divergence of other species of *Cyclura* (Colosimo, Knapp, Wallace, & Welch, 2014) and other species from Hispaniola (Brace et al., 2012; Gifford, Powell, Larson, & Gutberlet, 2004; Sly et al., 2010). Colosimo et al. (2014) found genetic differentiation as high as 27% for the Andros Island Rock Iguanas (*Cyclura cychlura cychlura*) and determined that iguanas may disperse passively (i.e., rafting) over water channels, which are prominent in the Andros Island landscape, but that migrations are unsuccessful over these water channels. Overall, many studies have shown clear evidence that Hispaniola Island landscape has important barriers which limits dispersal for many mobile animal species (Brace et al., 2012; Gifford et al., 2004; Sly et al., 2010), where Gifford et al. (2004) found up to 14% sequence divergence between northern and southern populations of a lizard species.

The congeners of *C. ricordii* examined to date typically reveal moderate to high levels of genetic structure. *Cyclura carinata*, the sister species to *C. ricordii*, has been studied extensively throughout the Turks and Caicos Islands (Bryan et al. 2007; Welch, 1997; Welch et al. 2004; Welch et al. 2017). Those populations are highly structured, and it is suspected that much of that structure predates their geographic isolation resulting from the rising sea levels of the current interglacial. Also, studies of *C. cychlura* in the Bahamas suggest a similar history of restricted gene flow (Aplasca et al. 2016; Colosimo et al., 2014; Malone et al. 2003). The work by Colosimo et al. (2014) provides perhaps the most appropriate comparison to demonstrate how geographic barriers can restrict gene flow for *Cyclura*. A different pattern is known for *Iguana delicatissima*, present in the Lesser Antilles, where little genetic structure among their populations was detected (Martin, Knapp, Gerber, Thorpe, & Welch, 2015).

### 4.1 Population genetic structure

Descriptive statistics for adults collected from Pedernales and Cabritos Island showed a significant excess of homozygotes in both populations. When the *F*_IS_ indices were generated for both hatchlings and adults in Pedernales, they were lower indicating that homozygote excess is lower in hatchlings than adults. However, the excess homozygosity and very small effective population size in adults may reflect sampling (i.e., small sample size, Pedernales = 20, Cabritos = 20). Hatchlings were collected from discrete nesting sites, and the assumption that these animals belong to the same population seems appropriate. Collection of adults was more haphazard. Many of the adults sampled were deceased as a result of predation by feral dogs. Because a precise origin of adult samples is unknown, we must accept that these samples may be more representative of a broader geographic range. Hence, independently of the origin of adult samples, the apparent elevated homozygosity may in part reflect small population size and a high degree of relatedness and could be a result of inbreeding, which could be a cause of concern and should be further evaluated.

### 4.2 Natal philopatry

We also analyzed the nesting behavior of *C. ricordii* using the same neutral molecular markers to test the hypothesis of natal philopatry. We focused on two nesting sites in Pedernales Province: Tierra and Malagueta. We found support for high levels of spatial clustering of nests within nesting sites consistent with a high return rate of females to nest in specific areas within these communal sites. The hypothesis of “natal philopatry,” however, was not supported when spatial and genetic data were combined because females nesting in the same cluster were no more closely related to each other than other females in different clusters. Further, the relationship between geographic distance and genetic distance among hatchlings within nesting sites was indicative of a slight fine‐scale genetic structuring.

Data on the genetic variation of hatchlings collected from Tierra and Malagueta showed an excess of homozygotes for both nesting sites, though it was only significant for Tierra. The homozygosity observed in hatchlings from Pedernales, along with elevated levels of homozygosity for adults, suggests that positive assortative mating may occur in the Pedernales population and that limited dispersal is present between nesting sites within the Pedernales geographic region. This may indicate that the breeding population that nests in Pedernales is too small. Natal philopatry in a small population may reinforce patterns of inbreeding among the adult population. Within nesting sites, fine‐scale genetic structuring observed for iguanas indicates that complete random mating is not present (*F*_ST_ = 0.019, *p* « 0.01; Table 5). This also suggests that some behavior related to the females' ability to choose a site to nest that increases her fitness should be expected. Given the threats that the Pedernales population faces, it is possible that the absence of an excess of heterozygotes in the hatchling dataset may be an artifact of small population size where nesting female relatedness is high, and recruitment among hatchlings is low.

Fine‐scale genetic structuring, within distances of 0.1–2 km, has been detected in a number of mobile animal species such as rattlesnakes (Clark, Brown, Stechert, & Zamudio, 2008; Gibbs, Prior, Weatherhead, & Johnson, 1997), carabid beetles (Brouat, Sennedot, Audiot, Leblois, & Rasplus, 2003), ungulates (Coltman, Pilkington, & Pemberton, 2003), and bush rats (Peakall, Ruibal, & Lindenmayer, 2003). Moore, Miller, Daugherty, and Nelson (2008) studied a long‐lived reptile (tuatara, *Sphenodon punctatus*) and found an overall genetic differentiation of 1.2% among subpopulations that were only 400 m apart (*R*_ST_ = 0.012, *p*‐val = 0.025). When the authors expanded their analysis to include a wider spatial range (750 m), the pattern disappeared (Moore et al., 2008). Moore et al. (2008) found that tuatara lack a philopatric behavior and concluded that long‐lived animals may present high genetic variation at a small scale without the presence of a complex social system. Female philopatry can reinforce fine‐scale genetic structuring, and this has been observed in several marine and terrestrial animals (Browne, Horrocks, & Abreu‐Grobois, 2010; Frantz, Hamann, & Klein, 2008; Hueter et al., 2005; Nussey et al., 2005). Very little is known about Ricord's Rock Iguana social system in Pedernales. Fine‐scale genetic structure may be a product of the sedentary nature of these lizards, the highly fragmented habitat in Pedernales, the scarcity of appropriate high‐quality nesting sites, and the constant threats that the species faces (Rupp, 2010). Similar results were found by Moore et al. (2008) for tuatara. The social system at the Pedernales nesting habitat may present complex intra‐ and interspecific interactions between males, which are known to present home range philopatry during the mating season, and females that tend to be philopatric toward a communal nesting site (Pérez‐Buitrago et al., 2010). The nuclear molecular markers used in this study did not provide the necessary resolution to fully discriminate sex‐specific differences. However, *R* ^2^ values for the Mantel's test were slightly positive. This highlights the importance of using appropriate markers that reflect dispersal patterns for both sexes if the complex nature of nesting in this genus is to be better understood. Sex‐biased dispersal is common in lizards (Gardner, Bull, Cooper, & Duffield, 2001; Stow, Sunnucks, Briscoe, & Gardner, 2001; Valenzuela & Janzen, 2001). Given the lack of variation observed for the mitochondrial markers tested (Arévalo et al., 1994), additional mitochondrial markers will have to be developed for this species before mtDNA sequence variation can be used as an effective tool for testing the natal philopatry hypothesis.

When spatial statistics were leveraged to test for natal philopatric behavior, a significant level of spatial clustering of nest sites at coarse and at fine spatial scales was supported through the years (2008–2013). However, this pattern varies through the years. The degree of clustering between nesting sites, at the coarse spatial scale, was expected due to the preexisting clustering of these communal nesting sites in Pedernales. However, when combined, spatial and genetic data do not support the return of related females to a specific nest aggregation within fondos. However, a strong fidelity toward specific areas, from females of unknown origin, within these nesting sites was supported with spatial data. Ripley's *K* and results from edge thinning techniques may support another alternative hypothesis. The "by‐product" hypothesis, which states that communal nesting results from a scarcity of nesting sites or other factors that cause coincidental aggregations of nesting mothers (Doody et al., 2009; Vitt, 1993), implies that the pattern detected using spatial statistic tools could hence be explained by the overall shortage of nest sites in Pedernales Province. However, an “adaptive” hypothesis may be considered, which states that fitness benefits to mothers, eggs, and hatchlings drive communal nesting (Doody et al., 2009). Where a suitable habitat for reproduction might be scarce and restricted to small areas, natural selection has favored individuals that return to the same natal areas to reproduce. Many reproductive advantages have been attributed to philopatric behaviors (Doody et al., 2009; Eckrich & Owens, 1995; Galef & Giraldeau, 2001; Giraldeau, 1997; Giraldeau, Valone, & Templeton, 2002; Robinson & Bider, 1988).

The amount of genetic variation detected among nests within clusters (*F*_SC_ = 0.204, *p* « 0.01 for Tierra and *F*_SC_ = 0.175, *p* « 0.01 for Malagueta) suggests that the patterns of aggregation observed with Ripley's *K* estimate are independent of the genetic structuring observed within each nesting site. These results are inconsistent with natal philopatry limiting dispersal for nest‐site selection within these nesting sites. Nests within each of the putative clusters are not more related to each other than the ones from other clusters. If natal philopatry were present, nests grouped within a single cluster should be more related to each other than nests from other clusters and variation among clusters should be higher and significant. Nevertheless, unrelated females may select the same nest site due to other environmental reasons, and elucidating these differences in relatedness may benefit from the gathering of ecological and behavioral data at the individual level for the Pedernales population. Further, the continued protection of these nesting sites since 2004 by an NGO, Grupo Jaragua, has led to the conservation of appropriate nesting habitat for multiple generations, and females may be returning to areas where increased protection facilitates nesting.

Imprinting is a concept that has been discussed for salmon and sea turtles (Lohmann, Putman, & Lohmann, 2008). Hatchlings may imprint on environmental parameters such as the type of soil and vegetation when they hatch. The southern nesting grounds for *C. ricordii* are characterized by low‐lying geological formations covered by fine, red argillic soil (Arias et al., 2004) and a rather uniform vegetation type where *Acacia* is predominant along with cacti. Given that significant gene flow between nesting sites was evident (Nm = 8.38), this indicates that natal philopatric behavior, if present, may not be strong or exclusive to finer spatial scales. This evidence could also be overcome by a more general “homing” behavior driven by other ecological factors such as imprinting on habitat features. The significant 1.9% of genetic differentiation between nesting sites within Pedernales shows how, even with a long‐lived reptile, very fine‐scale (<1.5 km) genetic structuring can be present and limited dispersal may be an outcome of natal philopatry for the Pedernales population. Changes in patterns of aggregations by year indicated that the observed patterns are not constant through space and time. Multiple factors may influence these yearly changes in the patterns of nest‐site aggregations. First, there is the possibility that females are not nesting every year (Iverson et al., 2004) and second, fluctuating environmental factors may be stimulating females to choose different locations over time. Tropical storms have a negative influence on hatching success (Iverson et al., 2004), and anthropomorphic disturbances may influence females when they are choosing locations to build nests and lay their eggs. These factors may bias female decision making, and as a result, nests may appear more spatially scattered. This might have been the case for the 2013 edge thinning results, where nests were more dispersed across both nesting sites than in previous years (Figures 7 and 8).

### 4.3 Conservation

The lack of philopatric behavior may have important consequences for future management decisions for *C. ricordii*. Multiple factors, such as quality of the nesting habitat, vegetation, and lack of good nesting patches, might influence recurrent use of specific sites within nesting areas by the same female (Bock et al., 1985; Knapp & Owens, 2008). Further exploration of the species' social system may be needed and highly variable sex‐specific markers should be developed to better test for natal philopatry at these sites. More conservation actions should be considered to improve the genetic viability of these populations. Extensions of the nesting habitats and translocation techniques have been discussed. Both techniques are not likely to be successful if animals reject their new homes. This would likely happen if this species reveals a more evident natal philopatric behavior.

Understanding nesting dynamics may be helpful to detect the most important nesting requirements for this endangered species. Several conservation actions have been taken in hopes of preventing further population declines for the species since 2002, and discussions regarding possible solutions in the future have started. Grupo Jaragua implemented the monitoring program for the species' nesting sites in Pedernales Province, and continues to actively work on habitat restoration and protection in Pedernales and along the southern shore of Enriquillo Lake. These actions have ensured the maintenance of these populations for the past 15 years.

The success and maintenance of these actions depends heavily on how much information we can gather about the nesting behavior of the species. Ecological parameters and intra‐ and interspecific interactions are extremely important if we want to translocate a population to a new environment or simply restore historic nesting grounds. Individuals confiscated from the illegal pet trade and from hunters may benefit from an understanding of the genetic structure of their populations. Hence, better‐informed management decisions for the reintroduction of these individuals to their populations of origin should be prioritized to avoid outbreeding among populations. Given the high degree of differentiation observed between the Cabritos Island and Pedernales populations, and the lack of gene flow between them, it may be prudent to maintain both populations as independent units for conservation purposes.

## CONFLICT OF INTEREST

None declared.

## **AUTHOR** **CONTRIBUTIONS**

Rosanna Carreras‐De León involved in all parts of the study. Stesha A. Pasachnik involved in the experimental design, acquisition of data (sampling), training of field techniques, submission of grants, manuscript edits, and financial support of fieldwork. Glenn P. Gerber involved in the experimental design, field training, grant submission, manuscript edits, and financial support of fieldwork. Christopher P. Brooks contributed to the design, analysis, and interpretation of the spatial analysis data. Ernst Rupp involved in the experimental design and provided nest data points for 6 years of his research (2008–2013) for the spatial analysis. Mark E. Welch involved in the experimental design, grant writing and submission, manuscript edits and revision, and financial support for the genetic analysis and training and served as an adviser.

## Data Availability

Microsatellite genotype data for all samples have been uploaded into the Dryad repository (https://doi.org/10.5061/dryad.bj3t434). The locations sampled for this study are cited in the reference section and shown in the map provided. Due to the sensitive nature of *Cyclura ricordii,* currently listed as Critically Endangered by the Red List of the IUCN and included in Appendix I of The Convention on International Trade in Endangered Species of Wild Fauna and Flora (CITES) specific geographic coordinates of nests and wild individuals will not be uploaded in the Dryad repository. All other data are provided in full in 3 section of this paper.
