# Supplementary figures and images for: Genetic structure at three spatial scales is consistent with limited philopatry in Ricord's Rock Iguanas (Cyclura ricordii)
Source: Ecol Evol. 2019 Jul 2;9(14):8331–50. doi: 10.1002/ece3.5414 (PMC6662429; doi:10.1002/ece3.5414)

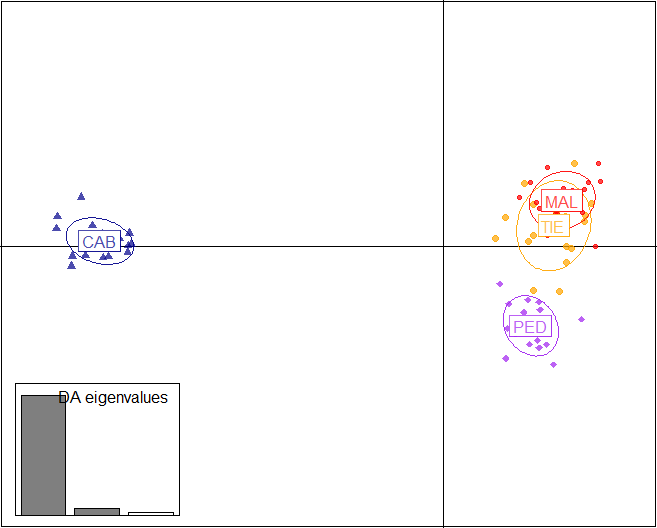

Supplement: Supplementary file 1 [file ECE3-9-8331-s001.tiff]
